# Relationship between DNA Mismatch Repair Deficiency and Endometrial Cancer

**DOI:** 10.4061/2011/256063

**Published:** 2011-12-08

**Authors:** Kenta Masuda, Kouji Banno, Megumi Yanokura, Yusuke Kobayashi, Iori Kisu, Arisa Ueki, Asuka Ono, Nana Asahara, Hiroyuki Nomura, Akira Hirasawa, Nobuyuki Susumu, Daisuke Aoki

**Affiliations:** Department of Obstetrics and Gynecology, Keio University School of Medicine, Shinanomachi 35 Shinjuku-Ku, Tokyo 160-8582, Japan

## Abstract

Some cases of endometrial cancer are associated with a familial tumor and are referred to as hereditary nonpolyposis colorectal cancer (HNPCC or Lynch syndrome). Lynch syndrome is thought to be induced by germline mutation of the DNA mismatch repair (MMR) gene. An aberration in the MMR gene prevents accurate repair of base mismatches produced during DNA replication. This phenomenon can lead to an increased frequency of errors in target genes involved in carcinogenesis, resulting in cancerization of the cell. On the other hand, aberrant DNA methylation is thought to play a key role in sporadic endometrial carcinogenesis. Hypermethylation of unmethylated CpG islands in the promoter regions of cancer-related genes associated with DNA repair leads to the cell becoming cancerous. Thus, both genetic and epigenetic changes are intricately involved in the process through which cells become cancerous. In this review, we introduce the latest findings on the DNA mismatch repair pathway in endometrial cancer.

## 1. Introduction

The incidence of endometrial cancer among malignant gynecological tumors has increased with lifestyle and environmental changes. In the US, 40,000 patients are diagnosed with endometrial cancer annually, and 7,500 patients die of this disease [1].

The number and prevalence of cases of endometrial cancer have increased worldwide and control of this cancer is urgently required. However, many aspects of the mechanism of carcinogenesis and pattern of advancement are unclear. Environmental factors such as obesity and a high estrogen level are thought to play important carcinogenic roles, but a close association with hereditary disposition has also been suggested, since double cancer and an increased incidence of cancer in relatives are common in patients with endometrial cancer.

Lynch syndrome, also known as hereditary nonpolyposis colorectal cancer (HNPCC), is a hereditary disease in which there is frequent development of colorectal, endometrial, and ovarian cancers. The cause is thought to be mutation of the DNA mismatch repair (MMR) gene in germ cells. However, the conventional explanation of the mechanism involving genetic changes—mutations of cancer-related genes—is inadequate and epigenetic changes in endometrial cancer are now being examined. In particular, aberrant DNA methylation is thought to play a key role in endometrial carcinogenesis. Breakdown of the DNA mismatch repair mechanism due to DNA hypermethylation plays a particularly important role in the development of endometrial cancer.

## 2. Lynch Syndrome

Lynch syndrome is a hereditary disease that includes frequent development of colorectal, endometrial, and ovar*i*an cancers, and which is inherited in an autosomal dominant manner. Lynch syndrome is caused by a hereditary defect in the DNA mismatch repair (MMR) gene and the incidences in colorectal and endometrial cancers are 2-3% and 1-2%, respectively [2]. This syndrome was initially reported by Wartin et al. in 1913 in a family with a high risk of development of colorectal cancer. Subsequent analysis of this family led Lynch to propose the disease concept of cancer family syndrome in 1971 [3, 4].

Six variants of the MMR gene, the causative gene in Lynch syndrome, have been cloned: *hMSH2*, *hMLH1*, *hMSH3*, *hMSH6*, *hPMS1*, and* hPMS2*. An aberration in one of these genes prevents accurate repair of base mismatches produced during DNA replication. In Lynch syndrome with an *hMLH1* or *hMSH2* mutation, the frequencies of colorectal and endometrial cancers are 68% and 62%, respectively, and the lifetime risk of developing endometrial cancer is higher than that for colorectal cancer in women [5].

Diagnosis of Lynch syndrome is based on clinical criteria. In 1990, the International Collaborative Group- (ICG-) HNPCC established the following diagnostic criteria for HNPCC, which are referred to as the classical Amsterdam criteria: (1) HNPCC is diagnosed when 3 or more patients with histologically confirmed colorectal cancer are present in a family line and one is a first relative of the other two; (2) colorectal cancer develops over two generations; (3) one case is diagnosed at younger than 50 years old [6]. In 1999, the new Amsterdam Criteria (Amsterdam II) [7] (Table 1) were published. These criteria address endometrial cancer, small intestinal cancer, urethral cancer, and kidney cancer, in addition to the colorectal cancer included in the classic criteria.

## 3. DNA Mismatch Repair Gene and Endometrial Cancer

DNA mismatch repair (MMR) system corrects DNA base pairing errors in newly replicated DNA. Mispaired nucleotides may be present after DNA replication, along with small insertion/deletion mutations that tend to occur at repetitive sequences. The MMR system is an excision/resynthesis system that can be divided into 4 phases: (i) recognition of a mismatch, (ii) recruitment of repair enzymes, (iii) excision of the incorrect sequence, and (iv) resynthesis by DNA polymerase using the parental strand as a template. This system is conserved through evolution from bacteria to human [8].

**Figure 1:**
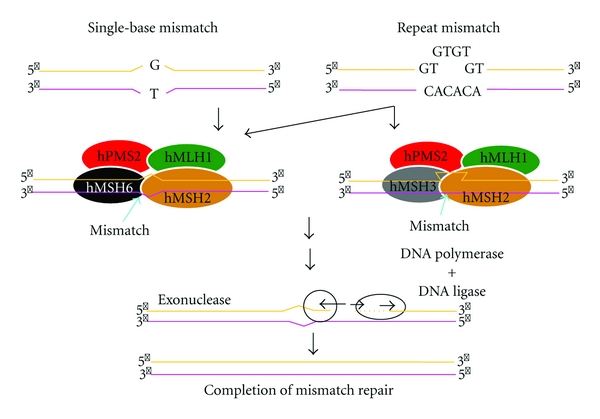


An aberration in one of MMR genes prevents accurate repair of base mismatches produced during DNA replication, resulting in production of a DNA chain of altered length, particularly in highly repeated sequences (microsatellites). This phenomenon is called microsatellite instability (MSI) and can lead to an increased frequency of errors in target genes involved in carcinogenesis, resulting in cancerization of the cell. Among the MMR genes, germline mutations of *hMLH1* on chromosome 3 and *hMSH2* on chromosome 2 are thought to cause most cases of HNPCC. Mutation of *hMSH6* has also been proposed to be important for development of HNPCC-associated endometrial cancer, but the details are unclear (Figure 1) [9].

## 4. Clinical Diagnostic Criteria for Lynch Syndrome

Since the Amsterdam Criteria for Lynch syndrome were proposed in 1991, several other diagnostic criteria, including the Japanese Criteria and the Bethesda Criteria, have been published. The confusion caused by the different criteria was resolved by revision of the Amsterdam Criteria by the ICG-HNPCC in 1999, to give the new Amsterdam Criteria [7] (Table 1). These criteria address endometrial cancer, small intestinal cancer, urethral cancer, kidney cancer, and colorectal cancer. Cases not meeting the classical Amsterdam Criteria may meet the new Amsterdam Criteria, and this has resulted in an increased number of cases diagnosed as Lynch syndrome. In addition, discovery of Lynch syndrome is now possible through investigation of familial histories of endometrial cancer patients. The revision also recognized the importance of cooperation among gynecologists for identification of Lynch syndrome. However, one concern with the new criteria is the omission of ovarian, breast, and stomach cancer, which may also be associated with Lynch syndrome.

The 1999 revised Amsterdam criteria II include endometrial cancer as a Lynch syndrome-related tumor, but women who develop endometrial cancer as the initial cancer and patients with a family tree with insufficient details are not included; thus, a high false negative rate has been reported based on these criteria [5]. For colorectal cancer, the Bethesda criteria require MSI testing, but this is not applicable for patients who develop endometrial cancer as the initial cancer. Thus, there is a need to establish criteria for selection of patients with endometrial cancer who should undergo screening [10].

## 5. Carcinoma of the Lower Uterine Segment and Lynch Syndrome

Endometrial cancer arises from the uterine body and fundus in many cases, but can also originate from the lower region of the uterine body through the upper region of the cervix. Such tumors are referred to as carcinoma of the lower uterine segment (LUS) or isthmus and account for 3–6.3% of all cases of endometrial cancer. The association of carcinoma of the LUS with Lynch syndrome has attracted recent attention. The frequency of Lynch syndrome in general endometrial cancer is 1-2% [2]. In contrast, Lynch syndrome has a high frequency in cases of carcinoma of the LUS, with one report in the US suggesting that 29% of such cases could also be diagnosed with Lynch syndrome and that the hMSH2 mutation was present at a high frequency in these cases [11]. Demonstration of an association between carcinoma of the LUS and Lynch syndrome in a large-scale survey would allow patients with carcinoma of the LUS to be classified as a high-risk group for Lynch syndrome [12].

## 6. Microsatellite Instability and Endometrial Cancer

Microsatellite instability occurs when the mismatch repair system is damaged. Microsatellites are DNA sequences of repeating units of 1 to 5 base pairs. Abnormalities in the mismatch repair system may cause replication errors in the repeating unit, leading to changes in length that are referred to as MSI. MSI caused by MMR gene aberration is detectable by PCR using microsatellite markers. In screening for Lynch syndrome, use of 5 microsatellite markers, two mononucleotide repeats (BAT26 and BAT25) and three dinucleotide repeats (D5S346, D2S123, and D17S250), is recommended [13]. MSI is observed in certain types of cancer, including 20 to 30% of cases of endometrial cancer [14]. These results suggest that MMR gene abnormalities occur frequently in endometrial cancer.

To investigate the status and characteristics of familial endometrial cancer, Banno et al. [15] surveyed the familial and medical histories of 385 patients who underwent treatment for endometrial cancer. MSI analysis was performed in 38 of these patients. The familial histories showed that 2 of the 385 cases met the new Amsterdam Criteria for Lynch syndrome, giving a rate of Lynch syndrome of about 0.5%. Investigation of familial accumulation of cancer in 890 relatives (439 men and 451 women) of the 38 endometrial cancer patients who underwent MSI analysis revealed high incidences of endometrial cancer, colorectal cancer, and ovarian cancer, suggesting that a hereditary factor common to Lynch syndrome is also involved in endometrial cancer. MSI analysis detected at least one of 5 microsatellite markers (D2S123, D3S1284, D5S404, D9S162, and hMSH2 intron 12) in 12 of the 38 cases (31.6%). This rate is very high compared to MSI in cancers of other organs, demonstrating that abnormal DNA mismatch repair plays an important role in endometrial cancer. The patients with MSI showed a tendency to have double cancer (such as ovarian cancer) compared with patients with microsatellite stability (MSS), although the difference was not significant (27% versus 15%). Regarding prognosis, none of the MSI-positive cases were fatal (0/11, 0%), while 5 MSI-negative (MSS) cases were fatal (5/27, 19%). The difference was not significant, but this tendency is similar to that for Lynch syndrome-associated colorectal cancer. The incidences of moderately differentiated adenocarcinoma G2 (36%) and poorly differentiated adenocarcinoma G3 (18%) tended to be higher in MSI-positive endometrial cancer, although again the difference was not significant. These findings appear contradictory with the favorable prognosis, but interestingly they may reflect the biological characteristics of endometrial cancer induced by abnormal DNA mismatch repair [16].

## 7. Screening for Endometrial Cancer and Prophylactic Hysterectomy in Lynch Syndrome

Women with Lynch syndrome have a high risk for endometrial cancer, with a life-long incidence of 40% to 60%, which is similar to or greater than that of colon cancer [17]. Therefore, a woman diagnosed with Lynch syndrome should undergo screening or prophylactic hysterectomy.

Potential screening methods include transvaginal ultrasound and endometrial biopsy. Transvaginal or transabdominal sonography is used to evaluate endometrial conditions and thickness. Some studies have shown a high false-positive rate and poor efficacy [18, 19], while others have shown high sensitivity and negative predictive values [20]. Endometrial biopsy is not used for general screening but may be useful for patients with Lynch syndrome with a high risk for endometrial cancer. Thus, women who have a DNA mismatch repair gene mutation or a family history of this mutation should undergo a biopsy every year at the age of 30–35 [21].

One article has reviewed 5 papers reporting results of gynecological cancer surveillance in Lynch syndrome. Of the five articles included in this review, three were retrospective observational study. One study was prospective cohort study in Finland. Another study was a prospective clinical study, which evaluated the performance of hysteroscopy and endometrial biopsy in women at risk of Lynch syndrome in France. This article concluded that, although surveillance can detect premalignant lesions, it does not completely remove the risk of invasive cancer and it remains unclear whether surveillance for gynecological cancer in women with Lynch syndrome would significantly decrease mortality [22].

Prophylactic hysterectomy has not been thought to reduce the cancer risk in women with Lynch syndrome. In 1997, the Cancer Genetics Studies Consortium suggested that there was insufficient evidence to recommend that women with Lynch syndrome should have prophylactic surgery to reduce the risk of gynecologic cancer [23]. However, prophylactic hysterectomy has been realistically conducted in some institutions. The effects of prophylactic hysterectomy are of interest. Schmeler et al. [24] showed that prophylactic hysterectomy had a cancer-protective effect based on a retrospective cohort analysis in 315 women with a detected hMLH1, hMSH2, or hMSH6 germline mutation from 1973 to 2004. Outcomes were compared between 61 patients who underwent hysterectomy for prophylaxis or benign disease and 210 patients who did not undergo prophylactic hysterectomy. None of the 61 patients in the hysterectomy group developed endometrial cancer, whereas 69 (33%) in the nonhysterectomy group had endometrial cancer. These results indicate that prophylactic hysterectomy significantly decreased the development of endometrial cancer.

These results suggest that further studies should be conducted to compare the morbidity and mortality between screening using sonography or endometrial biopsy and prophylactic surgery.

## 8. DNA Hypermethylation and Endometrial Cancer

**Figure 2:**
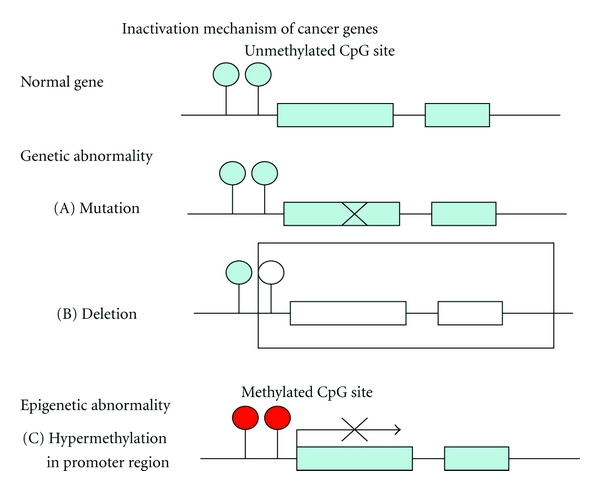


Epigenetics refers to the information stored after somatic cell division that is not contained within the DNA base sequence. Recent findings have shown that epigenetic changes—selective abnormalities in gene function that are not due to DNA base sequence abnormalities—play a significant role in carcinogenesis in various organs. In particular, the relationship between cancer and aberrant hypermethylation of specific genome regions has attracted attention. A completely new model for the mechanism of carcinogenesis has been proposed in which hypermethylation of unmethylated CpG islands in the promoter regions of cancer-related genes in normal cells silences these genes and leads to the cell becoming cancerous (Figure 2).

 The main difference between epigenetic abnormalities and genetic abnormalities such as gene mutations is that epigenetic changes are reversible and do not involve changes in base sequence. This suggests that restoration of gene expression is possible and that epigenetic data may lead to important molecular targets for treatment. Attempts have begun to detect aberrant DNA methylation of cancer cells present in minute quantities in biological samples and to apply the results to cancer diagnosis, prediction of the risk of carcinogenesis, and definition of the properties of a particular cancer. The MMR gene *hMLH1* is a typical gene that is silenced by DNA methylation. In endometrial cancer, *hMLH1* silencing is found in approximately 40% of cases and is an important step in the early stages of carcinogenesis, with the loss of DNA mismatch repair function proposed to lead to mutation of genes such as PTEN. In patients with endometrial cancer, Banno et al. found aberrant hypermethylation of *hMLH1*, APC, E-cadherin, and CHFR in 40.4%, 22.0%, 14.0%, and 13.3% of cases, respectively. A significant decrease in protein expression was found in patients with aberrant methylation of *hMLH1* (*P* < 0.01) and E-cadherin (*P* < 0.05), and aberrant methylation of *hMLH1* was also found in 14.3% of patients with atypical endometrial hyperplasia (AEH). However, no aberrant methylation of the four cancer-related genes was found in patients with a normal endometrium. These results indicate that aberrant methylation of specific genes associated with carcinogenesis in endometrial cancer does not occur in a normal endometrium. Aberrant methylation of *hMLH1* was most frequent, and the observation of this phenomenon in AEH, which is found in the first stage of endometrial cancer, supports the hypothesis that aberrant methylation of* hMLH1* is an important event in carcinogenesis in endometrial cancer [10, 25].

## 9. Conclusion

The DNA mismatch repair pathway is important in carcinogenesis of endometrial cancer. Recent analyses have shown that the MMR pathway can be impaired via both genetic and epigenetic mechanisms. Genetically, Lynch syndrome in cases of endometrial cancer is caused by a hereditary defect in the MMR gene. However, there have been fewer studies on endometrial cancer compared to colorectal cancer in patients with Lynch syndrome. Clarification of the pathology and development of screening and genetic tests are required for further progress in this area. Epigenetic research in endometrial cancer suggests that damage to the mismatch repair system plays a significant role in carcinogenesis and that DNA hypermethylation is important in this mechanism. Many attempts are currently being made to use epigenetic abnormalities as new methods of diagnosis and treatment based on control of methylation. Further studies of the genetic and epigenetic mechanisms may have potential for diagnosis, risk assessment, and treatment of endometrial cancer.

## Figures and Tables

**Table 1:** Clinical diagnostic criteria for HNPCC.

Amsterdam minimum criteria (1990)
(1) At least 3 cases of colorectal cancer in relatives (verified pathologically)
(2) One is a first degree relative of the other two
(3) At least two successive generations should be affected
(4) One case of colorectal cancer diagnosed before the age of 50 years old
(5) FAP should be excluded

Revised amsterdam criteria II (1998)

(1) At least 3 relatives with an HNPCC-associated cancer (cancer of the colorectum, endometrium, small bowel, ureter or renal pelvis)
(2–5) As for the minimum criteria
